# Left ventricular twist, but not circumferential-longitudinal shear angle, increases with increasing age in normal subjects

**DOI:** 10.1186/1532-429X-15-S1-P7

**Published:** 2013-01-30

**Authors:** Meral Reyhan, Ming Li, Himanshu Gupta, Steven G Llyod, Louis J Dell'Italia, Hyun J Kim, Thomas S Denney, Daniel Ennis

**Affiliations:** 1 Department of Radiological Sciences, University of California, Los Angeles, CA, USA; 2 Biomedical Physics Interdepartmental Program, University of California, Los Angeles, CA, USA; 3 Department of Electrical and Computer Engineering, Auburn University, Auburn, AL, USA; 4 Division of Cardiology, University of Alabama, Birmingham, AL, USA

## Background

Left Ventricular (LV) twist, defined as the difference in rotation between the apex and the base, has recently been suggested as a diagnostic imaging biomarker for LV dysfunction [1]. Increasing age is associated with an increase in apical rotation, which leads to an increase LV twist [2-4]. Recently, it has been suggested that LV circumferential-longitudinal shear angle (CL-shear angle) [5] may be a more robust imaging biomarker than LV twist, due to normalization within the formula for ventricular size and slice separation [6]. However, changes in CL-shear angle with respect to age have not been reported. CL-shear angle is defined as the difference between apical rotation times the epicardial radius of the apex and basal rotation times the epicardial radius of the base, divided by the distance between the apex and base. The purpose of this study was to evaluate age related changes in CL-shear angle.

## Methods

Normal subjects (n=54) with an age range of 20 to 70 years old (YO) were studied after obtaining informed consent. MRI was performed on a 1.5T scanner (Signa, GE Healthcare Milwaukee, WI) and grid tagged LV images were collect from the base to the apex [7]. LV twist and CL-shear angle measurements were derived from Fourier Analysis of STimulated echoes (FAST), a recently validated method for rapid quantification of LV twist [8]. The data was divided into five groups spanning each age by decade. Peak twist and peak CL-shear angle were compared for the five groups using a one-way ANOVA and Tukey's least significant difference (LSD) procedure for multiple comparisons.

## Results

Mean peak twist and CL-shear angle for the groups are summarized in Table 1. The one-way ANOVA of peak twist demonstrated differences among the different age groups (p=0.048). Further investigation with LSD, showed a significant difference between the 20-29 and 60-70 YO groups and between the 30-39 and 60-70 YO groups. However, the one-way ANOVA of peak shear-angle did not reveal any differences between the any of the groups (p=0.77).

**Table 1:** 

Age	Number of subjects (n)	Mean peak twist	Mean peak CL-shear angle	Apical epicardial radius	Basal epicardial radius	Apical rotation	Basal rotation	Distance
20-29 yrs	9	10.0±2.4°	4.7±1.5°	22.8±2.8 mm	29.7±6.0 mm	5.3±3.2°	-4.7±1.4°	5.6±0.7 cm
30-39 yrs	14	10.0±1.0°	5.0±1.0°	22.1±2.0 mm	31.7±3.2 mm	6.1±1.3°	-3.9±1.2°	5.0±0.3 cm
40-49 yrs	7	12.3±3.9°	5.0±1.2°	20.3±2.9 mm	32.0±3.7 mm	7.9±3.5°	-4.4±1.3°	5.6±0.7 cm
50-59 yrs	13	11.7±3.9°	5.1±1.6°	20.6±2.8 mm	31.7±2.9 mm	8.6±4.2°	-3.1±1.3°	5.2±0.7 cm
60-70 yrs	11	13.6±4.0°	5.6±2.0°	17.5±2.4 mm	28.2±2.5 mm	9.7±4.0°	-3.9±1.1°	5.0±0.5 cm
ANOVA P-value		P=0.048 20-29 and 30-39 are statistically different from 60-70	P=0.77 No difference	P=0.0002 60-70 statistically different from all other groups	P=0.09 No difference	P=0.02 20-29 and 30-39 are statistically different from 60-70 And 20-29 is statistically different from 50-59	P=0.07 No difference	P=0.03 20-29 is different from 30-39 and 60-70 40-49 is different from 30-39 and 60-70

## Conclusions

Peak LV twist has been shown to change with age in normal subjects, while peak CL-shear angle has demonstrated no significant change with age. The normalization of CL-shear angle over the different age groups despite an apparent increase in twist with age can largely be explained by an observed decrease in apical epicardial radius in older patients compared with younger patients. LV CL-shear angle may be a good biomarker for LV dysfunction in patients independent of age, unlike LV twist.

## Funding

Funding from AHA and UCLA to MLR and NIH to DBE.
